# Total Phenolics and Anthocyanins Contents and Antioxidant Activity in Four Different Aerial Parts of Leafy Sweet Potato (*Ipomoea batatas* L.)

**DOI:** 10.3390/molecules27103117

**Published:** 2022-05-12

**Authors:** Ruixue Jia, Chaochen Tang, Jingyi Chen, Xiongjian Zhang, Zhangying Wang

**Affiliations:** 1 Crops Research Institute, Guangdong Academy of Agricultural Sciences & Key Laboratory of Crop Genetic Improvement of Guangdong Province, Guangzhou 510640, China; jiaruixue91@126.com (R.J.); tangchaochen1988@163.com (C.T.); victerchen@163.com (J.C.); zhangxiongjian@gdaas.cn (X.Z.); 2 College of Agronomy and Biotechnology, Hebei Normal University of Science and Technology, Changli 066600, China

**Keywords:** leafy vegetable, radical scavenging, reducing power, color, antioxidant capacity

## Abstract

Leafy sweet potato (*Ipomoea batatas* L.) is an excellent source of nutritious greens and natural antioxidants, but reports on antioxidants content and activity at buds, leaves, petioles, and stems are scarce. Therefore, the total phenolics content (TPC), total anthocyanins content (TAC), and antioxidant activity (assessed by DPPH and ABTS radical scavenging activities and ferric reducing antioxidant power (FRAP)) were investigated in four aerial parts of 11 leafy sweet potato varieties. The results showed that varieties with pure green aerial parts, independently of the part analyzed, had higher TPC, FRAP, and ABTS radical scavenging activities. The green-purple varieties had a significantly higher TAC, while variety GS-17-22 had the highest TAC in apical buds and leaves, and variety Ziyang in petioles and stems. Among all parts, apical buds presented the highest TPC and antioxidant capacity, followed by leaves, petioles, and stems, while the highest TAC level was detected in leaves. The TPC was positively correlated with ABTS radical scavenging activity and FRAP in all parts studied, whereas the TAC was negatively correlated with DPPH radical scavenging activity. Collectively, the apical buds and leaves of sweet potato had the higher levels of nutritional values. These results would provide reference values for further breeding of leafy sweet potatoes.

## 1. Introduction

Sweet potato (*Ipomoea batatas* L.) is the sixth most significant crop in the world in terms of consumption because of its high yields and adaptability to various growing conditions [1]. The tuberous root of sweet potato is the main product, and it is widely used both as food and as raw material in starch production [2]. However, the leaves, petioles, and stems are underutilized in agro-processing and food industry [3]. The aerial parts of sweet potato are typically discarded in the field except for some use as livestock feed [4,5]. It is important to explore how the aerial parts of sweet potato can be utilized profitably [6].

The aerial parts of sweet potato are an excellent source of natural antioxidants [3,7,8], which are represented by phytochemicals such as phenolics and anthocyanins, and other components [9,10,11]. Phenolics are important because of their health-promoting physiological functions, including radical scavenging, anticancer, antibacterial, and anti-inflammatory actions [8,12,13]. Anthocyanins, which belong to a phenolic group, are bioactive components used in nutraceuticals [14]. Sweet potato greens contain much higher levels of polyphenols than many other major commercial vegetables, such as spinach, kale, broccoli, cabbage, and lettuce [10,15,16]. The predominant phenolics and anthocyanins in the leaves of sweet potato are caffeoylquinic acid derivatives and cyanidin 3-(6,6′-dicaffoyl-sophoroside)-5-glucoside, respectively [17,18]. In a latest study, the powder of the aerial parts (approximately 40 cm long at the tips) of sweet potato was able to replace 10% to 15% of the flour used in bread and could provide a significant health benefit as a functional product [7]. Therefore, the consumption of sweet potato greens as a source of antioxidants is recommended [8,19].

Sweet potato greens are often used as leafy vegetables in some tropical regions, especially in Southeast Asia [6,8,20,21]. Sweet potato is one of the few vegetables that can be grown during the monsoon season of the tropics and is the only vegetable greens available after a tsunami or typhoon [8]. Leafy sweet potato can be harvested several times during a growing season and is an alternative source of green leafy vegetables during the off-season, especially the humid months from May to August [22]. For these reasons and for meeting the local demand, leafy sweet potatoes are now cultivated in South China, and some new varieties with purple leaves are also beginning to be selected. Compared with the leaves of the common varieties studied earlier [21,23], the new varieties exhibit desirable characteristics for a leafy vegetable, such as tender leaves with no or very little pubescence and excellent edible quality. However, little is known about the total phenolics and anthocyanins contents and antioxidant properties of these leaf-specific sweet potatoes. Detailed reports on plant parts, including apical buds, petioles, and stems, are limited, although some previous research has focused on antioxidant content in the leaf and petiole parts [6,21,24]. The antioxidant contents and their activity in the sweet potato as a leafy vegetable need to be evaluated.

In addition to genotypes, color may be associated with the phenolics content and antioxidant activity in sweet potato leaves, stems, and tuberous root [22,25,26]. Yellow- and orange-fleshed sweet potatoes contain a blend of phenolic acids and have relatively high levels of carotenoids [3]. The purple-fleshed sweet potato has high levels of acylated anthocyanins and other phenolics with antioxidant and anti-inflammatory activities [27]. Isabelle et al. [28] suggested that dark green leafy and brightly colored vegetables tend to contain high levels of antioxidants. As with different flesh colors, sweet potatoes with different leaf colors grow under natural conditions. However, extensive research on antioxidant contents and property of leafy sweet potato with green and green-purple aerial parts has not been conducted.

Therefore, the objectives of this study were to: (1) investigate the total phenolics and anthocyanins contents and antioxidant activity at the terminal buds, leaves, petioles, and stems of 11 leafy sweet potato varieties; (2) compare the health benefits of the green and green-purple aerial parts of leafy sweet potatoes; and (3) determine the correlation between the contents of total phenolics and total anthocyanins and their antioxidant activity in four parts of the leafy sweet potato.

## 2. Results and Discussion

### 2.1. Agronomic Traits

**Figure 1:**
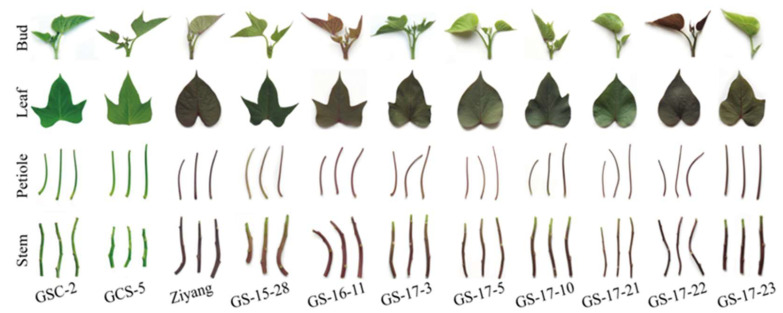
Buds, leaves, petioles, and stems of 11 leafy sweet potato varieties used in this study.

Seven agronomic traits of 11 leafy sweet potato varieties (Figure 1) are presented in Supplementary Table S1. As a result, all 11 leafy sweet potato varieties are semi-erect plant type. The leaf shape of variety GS-17-21 and Ziyang are cordate, GCS-5, GS-16-11, GS-17-3 and GS-17-10 are incised, while the other five varieties are acuminate-cordate. The color of vein, vine, and vine tip of GCS-5 and GSC-2 are pure green, whereas the other nine varieties are purple. In the present study, 11 leafy sweet potato varieties with no pubescence on their vine tips, indicating that these varieties were the ideal leafy sweet potatoes (Table S1).

### 2.2. Total Phenolics Content (TPC)

The TPC of apical buds, leaves, petioles, and stems in the 11 leafy sweet potato varieties are presented in Table 1. The TPC significantly varied among the varieties (*p* < 0.05). There was a large variation in the phenolics content among the sweet potato varieties and the different plant parts [29]. Among the varieties, GCS-5 had the highest TPC in four different parts, followed by GSC-2, whereas GS-17-3 and GS-17-21 had significantly lower TPC. Varieties (GSC-2 and GCS-5) with green aerial parts contained 1.91- to 3.04-fold higher (*p* < 0.05) TPC than varieties with green-purple aerial parts, including buds, leaves, petioles, and stems (Table 1). These results indicate that color is an important factor affecting the content of phenolic.

Phenolics are not distributed uniformly through the plant [30], and their distribution and content depend upon a set of factors, such as extraction technique, solvent, genotype, plant parts and environment [31,32,33]. The TPC was comparable to other studies that the TPC in leaves of two sweet potato varieties ranged from 59 to 357 mg GAE/100 g fw [28] and the TPC of 11 vegetables ranged from 33–152 mg GAE/100 g fw [34]. What’s more, we found that the buds contained the highest TPC, ranging from 65.32 to 248.22 mg GAE/100 g fw, followed by leaves (48.43 to 148.36 mg GAE/100 g fw), petioles (7.37 to 36.39 mg GAE/100 g fw), and stems (9.05 to 32.69 mg GAE/100 g fw). These results are consistent with the results of Ishida et al. [35], who reported that the TPC in sweet potato parts were in the order of leaves > stalks > stems.

### 2.3. Total Anthocyanins Content (TAC)

The effect of variety on TAC was significant at the *p* < 0.001 level (Table 1). GS-17-22 had the highest TAC in the apical buds (74.17 mg/100 g fw) and leaves (109.60 mg/100 g fw), whereas Ziyang exhibited the highest TAC in petioles (27.35 mg/100 g fw) and stems (13.88 mg/100 g fw). Averaged across varieties, leaves contained the highest TAC (42.21 mg/100 g fw), and this was 2.61, 3.96, and 6.78 times greater than in apical buds, petioles, and stems, respectively. However, different patterns were observed in five varieties (GS-17-3, GS-17-5, GS-17-10, GS-17-21, and GS-17-23), in which leaves had the highest TAC, followed by petioles, buds, and stems (Table 1). Kim et al. [25] reported that purple-fleshed sweet potatoes had a higher TAC in the roots, which ranged from 243 to 335 mg/100 g dry weight.

Dark-colored vegetables are known to be good sources of anthocyanins [36]. Chen et al. [37] reported that the sweet potato with purple leaves contained significantly higher levels of anthocyanins compared to green and yellow leaves. Our study results also showed that green-purple leafy sweet potatoes had significantly higher anthocyanins in each part than in the green leafy varieties.

### 2.4. Antioxidant Activities

Various methods can be used to evaluate the antioxidant activity of plant extracts, but no single standard is proposed because of the complexity of the extracts [19,38]. In the present study, three different methods, namely, the DPPH radical scavenging assay, ABTS radical scavenging activity, and FRAP assay were used to evaluate the antioxidant activity in the different parts of the 11 varieties. The antioxidant activity at each part, as assessed by the three methods, varied among the varieties. Varieties GCS-5, GS-17-3, and GS-17-21 had superior DPPH radical scavenging activity in four parts (Table 2). GCS-5 had the highest ABTS radical scavenging activity in buds, leaves, petioles, and stems, and the corresponding values were 36.33, 34.44, 18.68, and 16.42 μM TE/g fw, respectively (Table 3). The green-purple leafy variety Ziyang and the green leafy varieties (GSC-2 and GCS-5) had higher FRAP values, relative to the other varieties (Table 4). However, irrespective of the assessment method used, the results showed that the aerial parts of sweet potato had strong antioxidant activity. Truong et al. [39] also found that sweet potato leaf extracts had high DPPH radical scavenging activity with an average value of 38.1 μM TE/g fw in three commercial sweet potato cultivars. In addition, the aerial parts of sweet potato showed excellent antioxidant activity that exceeded the levels in other leafy vegetables [34,40,41,42].

In all of the antioxidant activity determinations, apical buds consistently had the highest levels, followed by leaves, petioles, and stems. Jang et al. [24] reported that leaves of sweet potato had higher antioxidant content and activity than petioles. In the present study, the antioxidant activity of each part in the green leafy varieties (GSC-2 and GCS-5) was stronger than in the green-purple leafy varieties. These findings are consistent with those of Isabelle et al. [28], who demonstrated that many dark green leafy vegetables had consistently high antioxidant activity and TPC.

### 2.5. Correlation between Antioxidant Activity and Total Phenolics and Total Anthocyanins Contents

Correlation analysis showed that TPC had a significantly (*p* < 0.05) positive correlation with antioxidant activity values from ABTS and FRAP assays in buds, leaves, petioles, and stems of leafy sweet potatoes (Table 5). However, the levels of TPC and DPPH radical scavenging activity were not correlated. The TAC in leaves, petioles, and stems had negatively significant correlation with DPPH radical scavenging activity, and a negative correlation was observed between TAC and ABTS radical scavenging activity in petioles (*p* < 0.05, Table 5). These findings are consistent with those of Xi et al. [15]. Li et al. [43] found that the antioxidant activity of highly pigmented vegetables, using the DPPH and FRAP assays, was correlated with the TPC, whereas TAC was only positively correlated with the FRAP value. Other studies reported that antioxidant activity was positively correlated with the TPC of leaves and roots [4,21,44] and the TAC of roots in sweet potato [45]. It appears that variety, climate, extraction methods, and plant part usage may all contribute to variations in antioxidant contents and activity and affect their correlations [46,47].

### 2.6. Cluster Analysis

**Figure 2:**
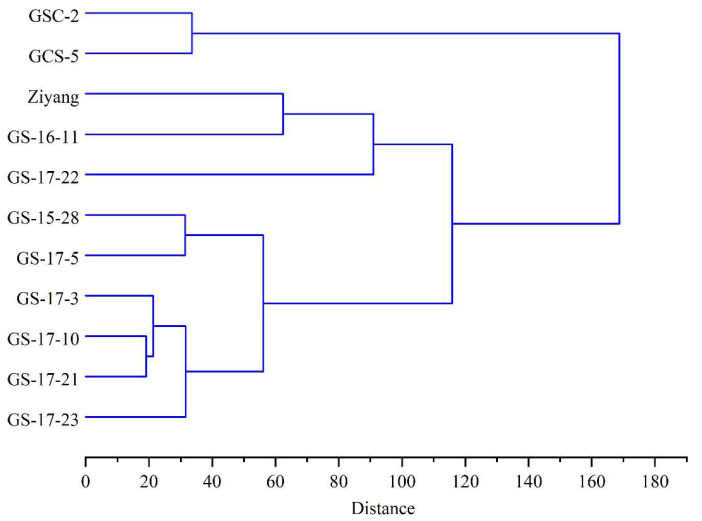
Cluster analysis among 11 leafy sweet potato varieties.

Cluster analysis was performed on the mean value to test the similarity among the different varieties based on TPC, TAC, DPPH, and ABTS radical scavenging activities and FRAP reducing power (Figure 2). Varieties GSC-2 and GCS-5 with pure green aerial parts were clustered together, and other nine varieties with green-purple parts were together. The result suggest that the color of leafy sweet potato is an important factor affecting TPC, TAC, and antioxidant activity.

## 3. Materials and Methods

### 3.1. Reagents

Acetonitrile and formic acid were HPLC grade and purchased from Merck (Darmstadt, HE, Germany). Standard Cyanidin 3-O-glucoside Chloride, 2,2-Diphenyl-1-picrylhydrazyl (DPPH), gallic acid (GAE), potassium ferricyanide, trichloroacetic acid, ferric chloride, and sodium carbonate were purchased from MACKLIN Biochemical Co., Ltd., (Shanghai, China). Hydrochloric acid, 95% ethanol, and hexane were obtained from Sinophorm Chemical Reagent Co., Ltd., (Shanghai, China). Sigma-Aldrich (Shanghai, China) supplied 6-hydroxy-2,5,7,8-tetramethylchromane-2-carboxylic acid (Trolox) and 2,2-azinobis-3-ethylbenzothiazoline-6-sulfonic acid diammonium salt (ABTS). Folin-Ciocalteu’s phenol reagent was obtained from Yuanye Biotechnology Co., Ltd., (Shanghai, China).

### 3.2. Plant Materials

Eleven leafy sweet potato varieties were used in this study. The varieties included GSC-2 and GCS-5 with green aerial parts and Ziyang, GS-15-28, GS-16-11, GS-17-3, GS-17-5, GS-17-10, GS-17-21, GS-17-22, and GS-17-23, which were bred with green-purple aerial parts. Cuttings of all of the 11 sweet potato varieties were planted on 4 August 2019 and grown using standard production practices [48] at the National Germplasm Guangzhou Sweet Potato Nursery, Guangdong Province, China (23°23′ N, 113°26′ E). The soil at the study site is clay loam with a pH of 5.81, organic matter content is 22.9 g kg^−1^, hydrolysable nitrogen content is 78.1 mg kg^−1^, available phosphorus content is 35.0 mg kg^−1^, and available potassium content is 173.0 mg kg^−1^ within the top 30 cm soil depth. The average monthly temperature of June, July and August is 28.6 °C, 29.8 °C and 30.0 °C, respectively. At 45 days after planting, the apical buds, one to four unfolded leaves (approximately 5.30 cm ×3.65 cm) from the top, and the corresponding petioles and stems were collected separately (Figure 1). Triplicate fresh samples of each part from each variety were immediately frozen in liquid nitrogen. All of the samples were ground using a liquid nitrogen grinder (A10 basic, IKA, Staufen, Germany) and stored at −80 °C until analysis.

### 3.3. Agronomic Traits Investigation

The agronomic traits of 11 leafy sweet potato varieties were investigated according to the description of Zhang and Fang [49], including plant type, parietal color, leaf shape, vein color, vine color, vine tip pubescence, and vine tip color.

### 3.4. Sample Extraction

Extraction of total phenolics and anthocyanins was conducted using the methods of Yang et al. [50] with some modifications. Two grams (g) of sample powder in a conical tube (50 mL) were transferred carefully to a 50 mL brown volumetric flask with a funnel carefully, and then 15 mL extraction solvent (95% alcohol) acidified with 1.5 N HCl (85:15, *v*/*v*) was added. The tube was rinsed, and the transfer was repeated twice until the tube was clean. Finally, the transfer objects were brought to 50 mL with the extraction solvent and soaked overnight at 4 °C. The supernatant was collected in a 50 mL conical tube, followed by centrifuging (5000 rpm, 10 min, 4 °C) with a centrifuge (ST16R, Thermo Scientific, Waltham, Massachusetts, USA). Another operation was used to remove polar lipids and other interfering compounds based on the methods of Song et al. [51]. Eighteen mL of hexane were added to 6 mL of crude extraction, and the tube was vigorously shaken before the hexane layer was removed. The operation was repeated five to six times until the hexane layer was completely removed. Extractions without chlorophyll were filtered with a 0.22 μM organic membrane and used for phenolics content, anthocyanins content, and antioxidant activity analysis.

### 3.5. Total Phenolics and Anthocyanins Determination

Total phenolic content (TPC) was quantified using the Folin–Ciocalteu method [52], with some modifications. One mL of sample extraction was diluted with water and mixed with 2 mL of Folin–Ciocalteu reagent. The mixture was maintained for 5 min, and then 2 mL of sodium carbonate (10 g/100 mL) was added. The reaction mixture was shaken and kept in darkness for 1 h at room temperature before being measured at 760 nm with an ultra-violet and visible spectrophotometer (DU800, Beckman Coulter). TPC was calculated using a gallic acid standard curve (*y* = 0.2802 *x* + 0.0605, *R*^2^ = 0.996) ranging from 1.045 to 10.45 μg/mL expressed as milligram gallic acid equivalent per 100 g of fresh weight (mg GAE/100 g fw).

Total anthocyanins content (TAC) was determined following the method described by Fuleki and Francis [53]. One-part sample with dark color were diluted 10 times with the solvent and stored in the darkness for 2 h to equilibrate the color. The total anthocyanins content was calculated using the following formula:

(1) $$\mathrm{TAC}\;\left(\frac{\mathrm{mg}}{100\;g}\right)={\mathrm{OD}}_{535}\times V\times N÷98.2÷m\times 100$$

where OD_535_, V, N, 98.2, and m were a spectrophotometric reading at 535 nm, extractive volume, dilution ratio, extinction coefficient value and sample weight, respectively.

### 3.6. DPPH Radical Scavenging Activity

The DPPH radical scavenging activity assay was performed following the procedure described by Sokolłetowska et al. [54] with some modifications. DPPH radical solution (200 μL of 0.2 mM) was added to a 50 μL aliquot of the 25-fold diluted extraction in a 96-well flat bottom microplate. After the mixture was mixed thoroughly and stored in the darkness for 20 min, the absorbance was measured using a multi-scan spectrum microplate reader (Thermo Scientific, Waltham, MA, USA) at 517 nm. A control containing 50 μL absolute ethanol was also included in each plate. The DPPH radical scavenging activity was calculated using Equation (2) with Trolox (0, 20, 40, 60, 80, 100, 120 and 140 μM), and results were expressed as μM Trolox equivalent (TE) per gram of fresh weight (μM TE/g fw).

(2) $$\mathrm{DPPH}\;\mathrm{radical}\;\mathrm{scavenging}\;\mathrm{rate}=\frac{\left(A_{1}-A_{0}\right)-\left(A_{i}-A_{j}\right)}{\left(A_{1}-A_{0}\right)}\times 100\%$$

where *A*_1_ and *A*_*i*_ represent the absorbance of solvent control and samples. *A*_0_ and *A*_*j*_ represent the absorbance of the blank control and a blank sample.

### 3.7. ABTS Radical Scavenging Activity

The ABTS radical scavenging activity assay was determined by the method of Re et al. [55] with slight modifications. Further details of the main experiment operation of ABTS assay have been described by Liao et al. [56]. Four μL extraction was added to 36 μL of absolute ethanol in a 96-well flat bottom microplate, then added 200 μL ABTS radical solution. After the mixture was mixed thoroughly and stored in darkness for 6 min, the absorbance was measured at 734 nm. The ABTS radical scavenging activity was calculated with Trolox (0–140 μM), and results were expressed as μM Trolox equivalent (TE) per gram of fresh weight (μM TE/g fw).

### 3.8. Ferric Reducing Antioxidant Power (FRAP) Assay

The FRAP assay was performed according to the method in Du et al. [57]. First, 1.0 mL of the 10-fold diluted extraction was mixed with 0.2 mL PBS and 1.5 mL 0.3% (*w*/*v*) potassium ferricyanide and incubated at 50 °C for 20 min. Then 1.0 mL of 10% (*w*/*v*) trichloroacetic acid was added and centrifuged for 10 min at 3000 r/min. After that, 2.0 mL of supernatant was taken and 0.5 mL 0.3% (*w*/*v*) ferric trichloride and 3.0 mL of distilled water was added. The absorbance of measured at 700 nm. The result was calculated by using a Trolox standard curve of 20 to 140 μM (*y* = 0.0144 *x* + 0.2627 and *R*^2^ = 0.9925) and expressed as μM TE/ g fw.

### 3.9. Statistical Analysis

Statistical analyses were performed using the SPSS 26.0 analytical software package (IBM, SPSS Inc., Chicago, IL, USA). The results were expressed as means ± one standard deviation of triplicate determinations and analyzed by one-way ANOVA. Duncan’s multiple range test was used to assess the multiple differences at the significance of *p* < 0.05. Cluster analysis based on mean values through Euclidean distance was used to reveal the similarity between varieties. The correlations between the total phenolics, total anthocyanins, and antioxidant activity were evaluated by the Pearson product moment coefficient of association. Student’s *t*-test was used to assess the significance of differences between green and green-purple parts at the *p* < 0.05 significance level.

## 4. Conclusions

Phenolics and anthocyanins are important functional components in sweet potato greens, and their antioxidant activity has a positive influence on human health. This study demonstrated that the sweet potato variety had a significant effect on the antioxidant levels and the properties of buds, leaves, petioles, and stems. Variety with pure green aerial parts had the higher TPC and antioxidant activity (assessed by FARP and ABTS assays) in four parts, whereas varieties with green-purple aerial parts possessed higher TAC. Apical buds consistently demonstrated the highest TPC and antioxidant capacity across in all varieties, followed by leaves, petioles, and stems. However, leaves contained the highest TAC, followed apical buds, petioles, and stems. ABTS radical scavenging activity and FRAP were significantly and positively correlated with TPC in four aerial parts, whereas the TAC was significantly and negatively correlated with DPPH radical scavenging activity in leaves, petioles and stems. In conclusion, the pure green varieties had higher TPC and greater antioxidant activity, whereas the green-purple varieties had higher levels of TAC. This study could be used by breeders for selectively increasing the antioxidant components of sweet potato greens.

## Figures and Tables

**Table 1:** Total phenolics content and total anthocyanins content of buds, leaves, petioles, and stems of 11 leafy sweet potato varieties.

Item	Total Phenolics Content (mg GAE/100 g fw)				Total Anthocyanins Content (mg/100 g fw)			
	Bud	Leaf	Petiole	Stem	Bud	Leaf	Petiole	Stem
Variety								
GSC-2	231.87 ± 13.57b	125.76 ± 2.36b	32.35 ± 3.17b	17.38 ± 2.36c	1.75 ± 0.08d	3.73 ± 0.06f	0.88 ± 0.01f	0.36 ± 0.11g
GCS-5	248.22 ± 14.85a	148.36 ± 1.36a	36.39 ± 1.29a	32.69 ± 1.12a	1.72 ± 0.45d	3.22 ± 0.06f	0.79 ± 0.07f	0.45 ± 0.07g
Ziyang	193.20 ± 2.58c	123.97 ± 4.46b	21.64 ± 1.01c	23.03 ± 0.52b	25.14 ± 0.85c	82.91 ± 2.68b	27.35 ± 0.92a	13.88 ± 0.99a
GS-15-28	132.98 ± 11.23d	53.19 ± 4.49fg	10.70 ± 0.78e	10.24 ± 1.18de	11.40 ± 0.93d	42.43 ± 3.31c	10.99 ± 0.37d	6.87 ± 0.11cd
GS-16-11	143.39 ± 5.15d	100.78 ± 5.57c	15.93 ± 1.27d	15.59 ± 1.18c	47.69 ± 0.53b	80.02 ± 4.73b	18.43 ± 1.58b	8.94 ± 0.31b
GS-17-3	65.32 ± 3.41f	56.17 ± 2.36fg	7.37 ± 0.45g	9.35 ± 0.45e	2.04 ± 0.12d	35.56 ± 2.80d	6.41 ± 0.92e	4.03 ± 0.26f
GS-17-5	109.93 ± 6.81e	69.25 ± 6.93de	9.21 ± 0.37fg	12.62 ± 0.93d	4.79 ± 0.11d	33.60 ± 2.33d	7.27 ± 0.06e	4.40 ± 0.04ef
GS-17-10	80.93 ± 3.41f	60.63 ± 3.09ef	8.62 ± 0.21fg	9.05 ± 1.12e	5.04 ± 0.03d	30.63 ± 0.14d	7.39 ± 0.21e	4.75 ± 0.07ef
GS-17-21	77.21 ± 4.64f	48.43 ± 1.36g	8.97 ± 1.62fg	11.43 ± 2.58de	2.65 ± 0.60d	20.99 ± 0.53e	6.81 ± 1.45e	5.99 ± 1.72de
GS-17-22	112.16 ± 10.54e	71.33 ± 7.93d	9.33 ± 0.10fg	11.58 ± 0.45de	74.17 ± 18.48a	109.60 ± 1.62a	16.68 ± 0.39c	9.04 ± 1.68b
GS-17-23	81.30 ± 1.58f	62.11 ± 8.94ef	9.92 ± 0.90f	9.35 ± 0.77e	4.64 ± 0.49d	44.04 ± 9.15c	10.01 ± 0.75d	7.79 ± 0.11bc
*p* value	<0.001	<0.001	<0.001	<0.001	<0.001	<0.001	<0.001	<0.001
**Color**								
Green	240.04 ± 15.56A	137.06 ± 12.50A	34.37 ± 3.10A	25.03 ± 8.55A	1.74 ± 0.29B	3.48 ± 0.30B	0.82 ± 0.07B	0.41 ± 0.09B
Green-purple	111.84 ± 39.87B	71.76 ± 24.28B	11.30 ± 4.45B	12.47 ± 4.40B	19.52 ± 25.30A	51.15 ± 27.32A	12.63 ± 7.07A	7.40 ± 3.13A
*p* value	<0.001	<0.001	<0.001	<0.001	<0.001	<0.001	<0.001	<0.001

The variety GSC-2 and GCS-5 with green aerial parts, and other nine varieties with green-purple aerial parts. For each variety, different lowercase letters (a, b, c, d, e, f, g) in a column indicate significant differences among variety means (*p* < 0.001). Different uppercase letters (A, B) in a column indicate significant differences among color means (*p* < 0.001).

**Table 2:** The 2,2-diphenyl-1-picrylhydrazyl (DPPH) radical scavenging assay of buds, leaves, petioles, and stems of 11 leafy sweet potato varieties.

Item	DPPH (μM TE/g fw)			
	Bud	Leaf	Petiole	Stem
Variety				
GSC-2	79.00 ± 1.23a	69.96 ± 1.64bc	70.31 ± 1.30ab	69.68 ± 1.35b
GCS-5	78.72 ± 0.38a	73.14 ± 4.78abc	70.29 ± 3.41ab	69.89 ± 3.69b
Ziyang	65.30 ± 2.58def	59.22 ± 0.95d	57.64 ± 2.57c	55.55 ± 2.89c
GS-15-28	59.45 ± 3.29f	60.36 ± 3.49d	57.98 ± 1.12c	59.49 ± 1.34c
GS-16-11	62.47 ± 4.84f	58.16 ± 0.82d	58.49 ± 1.73c	55.60 ± 9.54c
GS-17-3	76.25 ± 4.19ab	78.47 ± 1.68a	75.34 ± 0.54a	74.90 ± 3.06ab
GS-17-5	63.55 ± 2.73ef	59.51 ± 4.45d	59.63 ± 3.48c	58.53 ± 3.49c
GS-17-10	74.78 ± 2.20abc	74.18 ± 2.04abc	77.09 ± 10.66a	71.89 ± 4.64ab
GS-17-21	73.04 ± 4.30abc	73.34 ± 2.25abc	71.20 ± 3.82ab	77.84 ± 1.17a
GS-17-22	71.22 ± 8.19bcd	75.84 ± 3.84ab	65.10 ± 2.39bc	62.26 ± 1.48c
GS-17-23	69.11 ± 1.81cde	68.47 ± 5.82c	61.81 ± 0.93c	60.39 ± 5.40c
*p* value	<0.001	<0.001	<0.001	<0.001
**Color**				
Green	78.86 ± 0.83A	71.55 ± 3.64	70.30 ± 2.31	69.79 ± 2.49
Green-purple	68.24 ± 6.59B	67.51 ± 8.34	64.91 ± 8.34	64.05 ± 8.98
*p* value	<0.001	>0.05	>0.05	>0.05

The variety GSC-2 and GCS-5 with green aerial parts, and other nine varieties with green-purple aerial parts. For each variety, different lowercase letters (a, b, c, d, e, f) in a column indicate significant differences among variety means (*p* < 0.001). Different uppercase letters (A, B) in a column indicate significant differences among color means (*p* < 0.001).

**Table 3:** The 2,2-azinobis-3-ethylbenzothiazoline-6-sulfonic acid diammonium salt (ABTS) radical scavenging activity of buds, leaves, petioles, and stems of 11 leafy sweet potato varieties.

Item	ABTS (μM TE/g fw)			
	Bud	Leaf	Petiole	Stem
Variety				
GSC-2	33.49 ± 1.31a	30.98 ± 0.59ab	14.69 ± 1.55b	9.61 ± 2.12b
GCS-5	36.33 ± 0.22a	34.44 ± 1.50a	18.68 ± 0.89a	16.42 ± 2.55a
Ziyang	29.15 ± 0.04b	28.28 ± 0.60b	5.78 ± 0.68d	3.71 ± 0.48cd
GS-15-28	27.37 ± 1.55bc	21.33 ± 1.30c	3.48 ± 1.32e	4.73 ± 0.04cd
GS-16-11	28.75 ± 1.03b	27.53 ± 1.72b	5.95 ± 0.87d	5.93 ± 1.60cd
GS-17-3	24.13 ± 1.34cd	22.77 ± 0.69c	8.46 ± 2.23c	4.45 ± 1.18cd
GS-17-5	25.17 ± 0.19cd	19.59 ± 1.06c	4.18 ± 0.32de	3.19 ± 1.59d
GS-17-10	24.01 ± 0.59cd	20.32 ± 1.13c	2.91 ± 0.17e	5.15 ± 1.05cd
GS-17-21	23.29 ± 3.96d	19.56 ± 3.10c	3.57 ± 0.27e	6.86 ± 2.63bc
GS-17-22	24.17 ± 1.91cd	19.75 ± 2.82c	2.11 ± 0.07e	14.18 ± 2.82a
GS-17-23	19.23 ± 2.56e	22.88 ± 4.86c	2.29 ± 0.86e	5.51 ± 0.39cd
*p* value	<0.001	<0.001	<0.001	<0.001
**Color**				
Green	34.62 ± 1.81A	32.36 ± 2.08A	17.08 ± 2.40A	12.33 ± 4.22A
Green-purple	24.87 ± 3.30B	22.45 ± 3.76B	4.19 ± 2.02B	6.10 ± 3.51B
*p* value	<0.001	<0.001	<0.001	<0.01

The variety GSC-2 and GCS-5 with green aerial parts, and other nine varieties with green-purple aerial parts. For each variety, different lowercase letters (a, b, c, d, e) in a column indicate significant differences among variety means (*p* < 0.001). Different uppercase letters (A, B) in a column indicate significant differences among color means (*p* < 0.01).

**Table 4:** The ferric reducing antioxidant power (FRAP) assay of buds, leaves, petioles, and stems of 11 leafy sweet potato varieties.

Item	FRAP (μM TE/g fw)			
	Bud	Leaf	Petiole	Stem
Variety				
GSC-2	21.94 ± 2.20a	9.73 ± 0.99b	3.17 ± 0.07a	1.68 ± 0.13c
GCS-5	21.65 ± 3.14a	10.31 ± 0.69b	2.80 ± 0.16b	2.25 ± 0.03b
Ziyang	23.16 ± 3.45a	12.51 ± 1.58a	2.76 ± 0.39b	2.57 ± 0.05a
GS-15-28	10.25 ± 0.86bcd	4.17 ± 0.22de	0.30 ± 0.00f	0.47 ± 0.04d
GS-16-11	12.11 ± 0.63b	9.18 ± 0.40b	1.18 ± 0.35c	0.92 ± 0.17d
GS-17-3	8.46 ± 0.60cd	4.61 ± 0.39cde	0.65 ± 0.21def	0.70 ± 0.05e
GS-17-5	11.24 ± 1.56bc	5.76 ± 0.75c	0.90 ± 0.28cd	0.85 ± 0.08d
GS-17-10	10.83 ± 0.27bc	5.27 ± 0.09cd	0.62 ± 0.03def	0.69 ± 0.06e
GS-17-21	7.59 ± 0.76d	3.54 ± 0.09e	0.38 ± 0.10ef	0.96 ± 0.00d
GS-17-22	9.91 ± 0.10bcd	5.21 ± 0.27cd	0.77 ± 0.07de	0.90 ± 0.02d
GS-17-23	3.37 ± 0.44e	3.91 ± 0.28e	0.36 ± 0.12f	0.43 ± 0.05d
*p* value	<0.001	<0.001	<0.001	<0.001
**Color**				
Green	21.80 ± 2.43A	10.02 ± 0.82A	2.99 ± 0.23A	1.96 ± 0.32A
Green-purple	10.77 ± 5.24B	6.02 ± 2.88B	0.89 ± 0.77B	0.94 ± 0.63B
*p* value	<0.001	<0.01	<0.001	<0.001

The variety GSC-2 and GCS-5 with green aerial parts, and other nine varieties with green-purple aerial parts. For each variety, different lowercase letters (a, b, c, d, e, f) in a column indicate significant differences among variety means (*p* < 0.001). Different uppercase letters (A, B) in a column indicate significant differences among color means (*p* < 0.01).

**Table 5:** Correlation of antioxidant activity and total phenolics and total anthocyanins contents in buds, leaves, petioles, and stems from 11 leafy sweet potato varieties.

Item	Total Phenolics Content				Total Anthocyanins Content			
	Bud	Leaf	Petiole	Stem	Bud	Leaf	Petiole	Stem
DPPH	0.191	−0.190	0.044	−0.069	−0.335	−0.394 *	−0.589 **	−0.556 *
ABTS	0.885 **	0.853 **	0.909 **	0.429 *	−0.016	−0.212	−0.513 **	−0.340
FRAP	0.892 **	0.918 **	0.912 **	0.876 **	−0.066	0.105	0.062	0.088

Symbols * and ** indicate significant correlation at *p* < 0.05 and *p* < 0.01 levels, respectively.

## Data Availability

Exclude this statement.

## Supplementary Materials

The following supporting information can be downloaded at: https://www.mdpi.com/article/10.3390/molecules27103117/s1, Table S1: Agronomic traits of different leafy sweet potato varieties.

## Sample Availability

Samples of the Cyanidin 3-O-glucoside Chloride and gallic acid are available from the authors.

## References

[1] Lima P., CIP (2020). Discovery to Impact: Science-Based Solutions for Global Challenges.

[2] Dong J.U., Tai-Hua M.U., Sun H.N. (2017). Sweet potato and potato residual flours as potential nutritional and healthy food material. J. Integr. Agric..

[3] Wang S., Nie S., Zhu F. (2016). Chemical constituents and health effects of sweet potato. Food Res. Int..

[4] Cui L., Liu C., Li D., Song J. (2011). Effect of processing on taste quality and health-relevant functionality of sweet potato tips. Agric. Sci. China.

[5] Huang X., Tu Z., Xiao H., Li Z., Zhang Q., Wang H., Hu Y., Zhang L. (2013). Dynamic high pressure microfluidization-assisted extraction and antioxidant activities of sweet potato (*Ipomoea batatas* L.) leaves flavonoid. Food Bioprod. Process..

[6] Fu Z., Tu Z., Zhang L., Wang H., Wen Q., Huang T. (2016). Antioxidant activities and polyphenols of sweet potato (*Ipomoea batatas* L.) leaves extracted with solvents of various polarities. Food Biosci..

[7] Mau J., Lee C., Yang C., Chen R., Zhang Q., Lin S. (2020). Physicochemical, antioxidant and sensory characteristics of bread partially substituted with aerial parts of sweet potato. LWT.

[8] Islam S. (2006). Sweetpotato (*Ipomoea batatas* L.) leaf: Its potential effect on human health and nutrition. J. Food Sci..

[9] Cao G., Sofic E., Prior R.L. (1997). Antioxidant and prooxidant behavior of flavonoids: Structure-Activity relationships. Free Radic. Biol. Med..

[10] Lako J., Trenerry V.C., Wahlqvist M., Wattanapenpaiboon N., Sotheeswaran S., Premier R. (2007). Phytochemical flavonols, carotenoids and the antioxidant properties of a wide selection of Fijian fruit, vegetables and other readily available foods. Food Chem..

[11] Oki T., Masuda M., Furuta S., Nishiba Y., Suda I. (2010). Involvement of anthocyanins and other phenolic compounds in radical-scavenging activity of purple-fleshed sweet potato cultivars. J. Food Sci..

[12] Joseph S.V., Edirisinghe I., Burton-Freeman B.M. (2016). Fruit polyphenols: A review of anti-inflammatory effects in humans. Crit. Rev. Food Sci..

[13] Taira J., Taira K., Ohmine W., Nagata J. (2013). Mineral determination and anti-LDL oxidation activity of sweet potato (*Ipomoea batatas* L.) leaves. J. Food Compos. Anal..

[14] Khoo H.E., Azlan A., Tang S.T., Lim S.M. (2017). Anthocyanidins and anthocyanins: Colored pigments as food, pharmaceutical ingredients, and the potential health benefits. Food Nutr. Res..

[15] Xi L., Mu T., Sun H. (2015). Preparative purification of polyphenols from sweet potato (*Ipomoea batatas* L.) leaves by AB-8 macroporous resins. Food Chem..

[16] Karna P., Gundala S.R., Gupta M.V., Shamsi S.A., Pace R.D., Yates C., Narayan S., Aneja R. (2011). Polyphenol-rich sweet potato greens extract inhibits proliferation and induces apoptosis in prostate cancer cells in vitro and in vivo. Carcinogenesis.

[17] Zheng W., Clifford M.N. (2008). Profiling the chlorogenic acids of sweet potato (*Ipomoea batatas*) from China. Food Chem..

[18] Su X., Griffin J., Xu J., Ouyang P., Zhao Z., Wang W. (2019). Identification and quantification of anthocyanins in purple-fleshed sweet potato leaves. Heliyon.

[19] Morales-Soto A., García-Salas P., Rodríguez-Pérez C., Jiménez-Sánchez C., Cádiz-Gurrea M.D.L.L., Segura-Carretero A., Fernández-Gutiérrez A. (2014). Antioxidant capacity of 44 cultivars of fruits and vegetables grown in Andalusia (Spain). Food Res. Int..

[20] Sun H., Mu T., Xi L., Song Z. (2014). Effects of domestic cooking methods on polyphenols and antioxidant activity of sweet potato leaves. J. Agric. Food Chem..

[21] Xu W., Liu L., Hu B., Sun Y., Ye H., Ma D., Zeng X. (2010). TPC in the leaves of 116 sweet potato (*Ipomoea batatas* L.) varieties and Pushu 53 leaf extracts. J. Food Compos. Anal..

[22] Li M., Jang G.Y., Lee S.H., Kim M.Y., Hwang S.G., Sin H.M., Kim H.S., Lee J., Jeong H.S. (2017). Comparison of functional components in various sweet potato leaves and stalks. Food Sci. Biotechnol..

[23] Sun H., Mu T., Xi L., Zhang M., Chen J. (2014). Sweet potato (*Ipomoea batatas* L.) leaves as nutritional and functional foods. Food Chem..

[24] Jang Y., Koh E. (2019). Antioxidant content and activity in leaves and petioles of six sweet potato (*Ipomoea batatas* L.) and antioxidant properties of blanched leaves. Food Sci. Biotechnol..

[25] Kim J.M., Park S.J., Lee C.S., Ren C., Kim S.S., Shin M. (2011). Functional properties of different Korean sweet potato varieties. Food Sci. Biotechnol..

[26] Steed L.E., Truong V.D. (2008). Anthocyanin content, antioxidant activity, and selected physical properties of flowable purple-fleshed sweetpotato purees. J. Food Sci..

[27] Grace M.H., Yousef G.G., Gustafson S.J., Truong V., Yencho G.C., Lila M.A. (2014). Phytochemical changes in phenolics, anthocyanins, ascorbic acid, and carotenoids associated with sweetpotato storage and impacts on bioactive properties. Food Chem..

[28] Isabelle M., Lee B.L., Lim M.T., Koh W., Huang D., Ong C.N. (2010). Antioxidant activity and profiles of common vegetables in Singapore. Food Chem..

[29] Jung J., Lee S., Kozukue N., Levin C.E., Friedman M. (2011). Distribution of phenolic compounds and antioxidative activities in parts of sweet potato (*Ipomoea batata* L.) plants and in home processed roots. J. Food Compos. Anal..

[30] Padda M.S., Picha D.H. (2007). Antioxidant activity and phenolic composition in ‘Beauregard’ sweetpotato are affected by root size and leaf age. J. Am. Soc. Hortic. Sci..

[31] Ahmed Z., Hassan S.E., Fatima M., Mahdi C., Chaqroune A., Taleb M. (2021). Effects of extraction technique and solvent on phytochemicals, antioxidant, and antimicrobial activities of cultivated and wild rosemary (*Rosmarinus officinalis* L.) from Taounate Region (Northern Morocco). Biointerface Res. Appl. Chem..

[32] Zeroual A., Sakar E.H., Eloutassi N., Mahjoubi F., Chaouch M., Chaqroune A. (2021). Wild chamomile [*Cladanthus mixtus* (L.) Chevall.] collected from central-northern Morocco: Phytochemical profiling, antioxidant, and antimicrobial activities. Biointerface Res. Appl. Chem..

[33] Sakar E.H., El Yamani M., Boussakouran A., Ainane A., Ainane T., Gharby S., Rharrabti Y. (2021). Variability of oil content and its physicochemical traits from the main almond [*Prunus dulcis* Mill. Webb, D.A.] cultivars grown under contrasting environments in north-eastern Morocco. Biocatal. Agric. Biotechnol..

[34] Andarwulan N., Batari R., Sandrasari D.A., Bolling B., Wijaya H. (2010). Flavonoid content and antioxidant activity of vegetables from Indonesia. Food Chem..

[35] Ishida H., Suzuno H., Sugiyama N., Innami S., Tadokoro T., Maekawa A. (2000). Nutritive evaluation on chemical components of leaves, stalks and stems of sweet potatoes (*Ipomoea batatas* poir). Food Chem..

[36] Kongkachuichai R., Charoensiri R., Yakoh K., Kringkasemsee A., Insung P. (2015). Nutrients value and antioxidant content of indigenous vegetables from Southern Thailand. Food Chem..

[37] Chen S.P., Wang S.Y., Huang M.Y., Lin K.H., Hua S.M., Lu H.H., Lai Y.C., Yang C.M. (2018). Physiological and molecular analyses of chlorophyllase in sweet potatoes with different-colored leaves. S. Afr. J. Bot..

[38] Ciž M., Cižova H., Denev P., Kratchanova M., Slavov A., Lojek A. (2010). Different methods for control and comparison of the antioxidant properties of vegetables. Food Control.

[39] Truong V.D., Mcfeeters R.F., Thompson R.T., Dean L.L., Shofran B. (2007). Phenolic acid content and composition in leaves and roots of common commercial sweetpotato (*Ipomea batatas* L.) cultivars in the United States. J. Food Sci..

[40] Cömert E.D., Mogol B.A., Gökmen V. (2020). Relationship between color and antioxidant capacity of fruits and vegetables. Curr. Res. Food Sci..

[41] Deng G., Lin X., Xu X., Gao L., Xie J., Li H. (2013). Antioxidant capacities and total phenolic contents of 56 vegetables. J. Funct. Foods.

[42] Gunathilake K.D.P.P., Ranaweera K.K.D.S. (2016). Antioxidative properties of 34 green leafy vegetables. J. Funct. Foods.

[43] Li H., Deng Z., Zhu H., Hu C., Liu R., Young J.C., Tsao R. (2012). Highly pigmented vegetables: Anthocyanin compositions and their role in antioxidant activities. Food Res. Int..

[44] Zhu F., Cai Y., Yang X., Ke J., Corke H. (2010). Anthocyanins, hydroxycinnamic acid derivatives, and antioxidant activity in roots of different chinese purple-fleshed sweetpotato genotypes. J. Agric. Food Chem..

[45] Gan L.J., Yang D., Shin J.A., Kim S.J., Hong S.T., Lee J.H., Sung C.K., Lee K.T. (2012). Oxidative comparison of emulsion systems from fish oil-based structured lipid versus physically blended lipid with purple-fleshed sweet potato (*Ipomoea batatas* L.) extracts. J. Agric. Food Chem..

[46] Anastácio A., Carvalho I.S. (2013). Spotlight on PGI sweet potato from Europe: Study of plant part, time and solvent effects on antioxidant activity. J. Food Biochem..

[47] Kuan L., Thoo Y., Siow L. (2016). Bioactive components, ABTS radical scavenging capacity and physical stability of orange, yellow and purple sweet potato (*Ipomoea batatas*) powder processed by convection- or vacuum-drying methods. Int. J. Food Sci. Technol..

[48] Chen J., Fang B., Li Y., Zhang X., Wang Z., Huang L., Luo Z., Chen X. (2013). Breeding of a new variety Guang Cai Shu No.3 for the tips of sweet potato vine as vegetables. Guangdong Agric. Sci..

[49] Zhang Y., Fang B. (2006). Descriptors and Data Standard for Sweetpotato [Ipomoea batatas (L.) Lam.].

[50] Yang Z., Chen Z., Yuan S., Zhai W., Piao X., Piao X. (2009). Extraction and identification of anthocyanin from purple corn (*Zea mays* L.). Int. J. Food Sci. Technol..

[51] Song J., Li D., Liu C., Zhang Y. (2011). Optimized microwave-assisted extraction of total phenolics (TP) from *Ipomoea batatas* leaves and its antioxidant activity. Innov. Food Sci. Emerg..

[52] Yu Y., Xu Y., Wu J., Xiao G., Fu M., Zhang Y. (2014). Effect of ultra-high pressure homogenisation processing on phenolic compounds, antioxidant capacity and anti-glucosidase of mulberry juice. Food Chem..

[53] Fuleki T., Francis F.J. (1968). Quantitative Methods for Anthocyanins.

[54] Sokół-Betowska A., Kucharska A.Z., Winska K., Szumny A., Nawirska-Olszanska A., Mizgier P., Wyspianska D. (2014). Composition and antioxidant activity of red fruit liqueurs. Food Chem..

[55] Re R., Pellegrini N., Proteggente A., Pannala A., Yang M., Rice-Evans C. (1999). Antioxidant activity applying an improved ABTS radical cation decolorization assay. Free Radic. Biol. Med..

[56] Liao M., Zou B., Chen J., Yao Z., Huang L., Luo Z., Wang Z. (2019). Effect of domestic cooking methods on the anthocyanins and antioxidant activity of deeply purple-fleshed sweetpotato GZ9. Heliyon.

[57] Du G., Li M., Ma F., Dong L. (2009). Antioxidant capacity and the relationship with polyphenol and Vitamin C in Actinidia fruits. Food Chem..

