# Supplementary material for: Total Phenolics and Anthocyanins Contents and Antioxidant Activity in Four Different Aerial Parts of Leafy Sweet Potato (Ipomoea batatas L.)
Source: Molecules. 2022 May 12;27(10):3117. doi: 10.3390/molecules27103117 (PMC9146295; doi:10.3390/molecules27103117)
Supplement: Supplementary file 1 [file molecules-27-03117-s001.zip › molecules-1721209-supplementary.pdf]

Supplementary Material

Table S1. Agronomic traits of different leafy sweet potato varieties.

variety	Plant type	Parietal color	Leaf shape	Vein color	Vine color	Vine tip pubes-ence	Vine tip color
GSC-2	semi-erect	green	acuminate-cor-date	green	green	none	green
GCS-5	semi-erect	green	incised	green	green	none	green
Ziyang	semi-erect	purple	cordate	purple	purple	none	purple
GS-15-28	semi-erect	light purple	acuminate-cor-date	purple	purple	none	purple
GS-16-11	semi-erect	purple	incised	purple	purple	none	purple
GS-17-3	semi-erect	yellow green	incised	purple	purple	none	purple
GS-17-5	semi-erect	yellow green	acuminate-cor-date	purple	purple	none	purple
GS-17-10	semi-erect	light purple	incised	purple	purple	none	purple
GS-17-21	semi-erect	light purple	cordate	purple	purple	none	purple
GS-17-22	semi-erect	purple	acuminate-cor-date	purple	purple	none	purple
GS-17-23	semi-erect	light green	acuminate-cor-date	purple	purple	none	purple
